# Mitochondrial DNA mutations and respiratory chain dysfunction in idiopathic and connective tissue disease-related lung fibrosis

**DOI:** 10.1038/s41598-019-41933-4

**Published:** 2019-04-02

**Authors:** Veronika K. Jaeger, Dirk Lebrecht, Andrew G. Nicholson, Athol Wells, Harshil Bhayani, Amiq Gazdhar, Michael Tamm, Nils Venhoff, Thomas Geiser, Ulrich A. Walker

**Affiliations:** 1 Department of Rheumatology, University Hospital Basel, Basel, Switzerland; 2 Department of Rheumatology and Clinical Immunology, Medical Center - University of Freiburg, Faculty of Medicine, University of Freiburg, Freiburg, Germany; 3 Department of Pediatrics and Adolescent Medicine, Division of Pediatric Hematology and Oncology, Medical Center, Faculty of Medicine, University of Freiburg, Freiburg, Germany; 4 Department of Histopathology, Royal Brompton Hospital, Royal Brompton and Harefield NHS Foundation Trust, London, UK; 5 National Heart and Lung Institute, Imperial College, London, UK; 6 Interstitial Lung Disease Unit, Royal Brompton Hospital, Royal Brompton and Harefield NHS Foundation Trust, London, UK; 7 Royal Brompton and Harefield NHS Foundation Trust, London, UK; 8 Department of Pulmonary Medicine, University Hospital Bern, Bern, Switzerland; 9 Department of Pneumology, University Hospital Basel, Basel, Switzerland

## Abstract

Reactive oxygen species (ROS) are implicated in the aetiology of interstitial lung disease (ILD). We investigated the role of large-scale somatically acquired mutations in mitochondrial DNA (mtDNA) and consecutive respiratory chain dysfunction as a trigger of ROS-formation and lung fibrosis. Mitochondria were analysed in lung biopsies from 30 patients with idiopathic or connective tissue disease (CTD)-related ILD and 13 controls. In 17 patients we had paired biopsies from upper and lower lobes. Control samples were taken from lung cancer resections without interstitial fibrosis. Malondialdehyde, a marker of ROS-formation, was elevated in ILD-biopsies (p = 0.044). The activity of the mitochondrial respiratory chain (cytochrome c-oxidase/succinate dehydrogenase [COX/SDH]-ratio) was depressed in ILD (median = 0.10,) compared with controls (0.12, p < 0.001), as was the expression of mtDNA-encoded COX-subunit-2 protein normalized for the nucleus-encoded COX-subunit-4 (COX2/COX4-ratio; ILD-median = 0.6; controls = 2.2; p < 0.001). Wild-type mtDNA copies were slightly elevated in ILD (p = 0.088). The common mtDNA deletion was only present at low levels in controls (median = 0%) and at high levels in ILD (median = 17%; p < 0.001). In ILD-lungs with paired biopsies, lower lobes contained more malondialdehyde and mtDNA deletions than upper lobes and had lower COX2/COX4-ratios and COX/SDH-ratios (all p < 0.001). Acquired mtDNA-mutations and consecutive respiratory chain dysfunction may both trigger and perpetuate ROS-formation in ILD.

## Introduction

Interstitial lung disease (ILD) can be idiopathic, or occur in conjunction with systemic sclerosis (SSc) and other connective tissue diseases (CTD). A current hypothesis is that ILD results from a sequential ‘multi-hit’ injury to lung epithelial cells and that incomplete repair fosters the release of transforming growth factor (TGF) *β* and other profibrotic mediators, ultimately resulting in the continued deposition of excess extracellular matrix and collagen^1^. The notion of a crucial involvement of the immune system in the pathogenesis of lung fibrosis is challenged by the observation that ILD is in clinical practice not amenable to immunosuppression and that many patients have remarkably little cellular inflammation in affected lungs^2,3^.

Reactive oxygen species (ROS) in contrast may play an important role in the pathogenesis of idiopathic and CTD-related ILD. ROS are present at great abundance early in the disease evolution and are constitutively produced by pulmonary fibroblasts in ILD-patients^4,5^. The mechanism that initiates the generation of hydrogen peroxide and superoxide in CTD resides within the fibroblasts and is independent of cytokines^6^. Oxidant stress also enhances fibroblast proliferation and collagen formation^6^, possibly by activating latent TGFβ1, a process that again promotes ROS formation^7–9^. This TGFβ1-cycle is responsible for the expression of genes associated with the epithelial mesenchymal transition i.e. the transformation of pulmonary epithelial cells to fibroblasts and myofibroblasts^8,10,11^. Interestingly, the precise source of ROS in human ILD is unknown.

Mitochondria may play an important role in the pathogenesis of ILD as firstly, they are the main source of ROS producers within cells and secondly are in turn themselves prone to an oxidative injury of their membranes, proteins and DNA, e.g. mitochondrial DNA (mtDNA). The mitochondrial genome is particularly predisposed to oxidative damage due to its proximity to the mitochondrial respiratory chain, its lack of protective histones and limited DNA repair mechanism^12^. Unopposed mtDNA mutagenesis by endogenous ROS may interfere with the correct synthesis of mitochondrial RNA templates for the mitochondrial respiratory chain protein subunits. The resulting respiratory chain dysfunction could then promote ROS formation, with ROS then either attacking the respiratory chain itself, or in turn damaging mtDNA^12^. ROS may therefore close a vicious circle consisting of entwined respiratory chain insults and mtDNA mutations. Mitochondrial ROS as potent drivers of TGFβ1 activation may then also contribute to the relentless progression of ILD^6,8,13^.

We have recently discovered enhanced mtDNA mutagenesis, ROS-formation and mtDNA-encoded respiratory chain dysfunction in bleomycin induced pulmonary fibrosis^14^. The aim of this investigation was therefore to study, if mitochondrial lesions are also associated with ROS-formation and the pathogenesis of ILD in humans.

## Results

### Patients and lung biopsies

This study is based on a total of 60 lung biopsies; 47 biopsies (24 from the upper lobe and 23 from the lower lobe) were from 30 adult ILD-patients. From 17 patients (Eleven CTD and five idiopathic ILD-patients, one unknown) we had paired biopsies from two different lobes. In 13 patients (four CTD and five idiopathic ILD-patients) only one sample was available for analysis; two patients had a biopsy from the upper lobe, eleven patients from the lower lobe.

Thirteen biopsies were obtained from 13 controls; seven biopsies were from the upper lobes and six from the lower lobes.

Fifteen ILD-patients (50%) had a CTD (seven of which had SSc) and 14 patients (47%) had idiopathic ILD (Table 1). In one ILD-patient (3%) this information was unavailable. Of note, controls were significantly older and more frequently current smokers than ILD-patients.

**Table 1:** Demographics and clinical characteristics of the study population and the study results of their lung biopsies.

Characteristics		Controls	All ILD*	CTD-related ILD	Idiopathic ILD	p-value (controls *vs* all ILD)
**n patients**		**13**	**30**	**15**	**14**	
Female sex		62	47	67	29	0.37
Age, mean years (SD)		67.4 (13.7)	50.0 (11.2)	49.1 (10.7)	51.0 (12.6)	<0.001
Smoking behaviour	Never	10	44	50	36	0.16
	Previous	70	48	50	46	
	Current	20	8	0	18	
FVC, % of predicted		101 (90–105)	84 (67–88)	85 (69–89)	83 (64–85)	<0.001
**n biopsies**		**13**	**47**	**26**	**19**	
MDA, μmol/g lung tissue		91 (87–101)	101 (91–131)	105 (90–133)	101 (96–117)	0.044
COX/SDH-ratio		0.12 (0.11–0.18)	0.10 (0.04–0.13)	0.10 (0.04–0.13)	0.10 (0.04–0.12)	<0.001
mtDNA, copies/pulmonary cell		315 (267–471)	419 (323–527)	367 (301–484)	434 (365–542)	0.088
COX2/COX4-ratio		2.18 (2.10–2.43)	0.64 (0.51–0.89)	0.62 (0.51–0.89)	0.72 (0.53–0.89)	<0.001
common deletion, % of mtDNA molecules		0 (0–13)	17 (0–32)	21 (0–36)	15 (0–29)	<0.001

Presented are either percent or median (interquartile range) if not otherwise specified.

COX2/COX4, cytochrome c-oxidase subunit 2 protein normalized for cytochrome c-oxidase subunit 4 protein; COX/SDH-ratio, cytochrome c-oxidase/ succinate dehydrogenase activity ratio; CTD, connective tissue disease; ILD, interstitial lung disease; MDA, malondialdehyde; mtDNA, mitochondrial DNA; FVC, forced vital capacity; SD, standard deviation.

*In one patient it is unknown if the patient suffered from a CTD-related ILD or an idiopathic IDL.

### Mitochondrial function and ROS-formation in all lung biopsies

We compared the 47 lung biopsies from the ILD-patients with the 13 biopsies from controls (Table 1), the median pulmonary MDA content was slightly elevated in biopsies from ILD-patients (p = 0.044).

**Figure 1:**
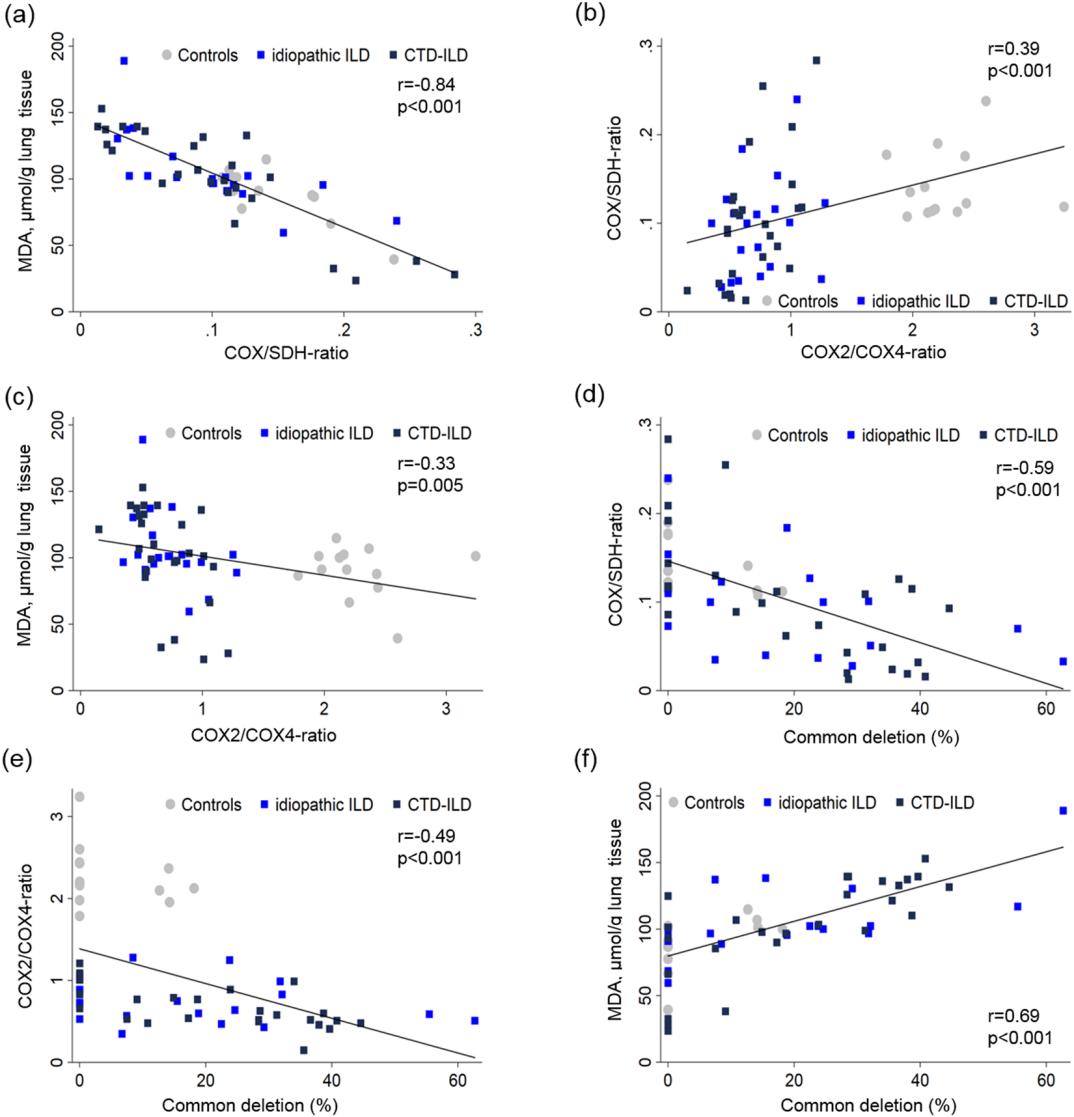
Correlations between pulmonary levels of malondialdehyde (MDA) as a surrogate of ROS formation, low COX/SDH-activity ratios as a marker of impaired mtDNA-encoded respiratory chain function, low COX2/COX4-ratios as a measure of impaired mtDNA encoded protein synthesis, and the common mtDNA deletion as a marker of mtDNA mutagenesis. Depicted are all lung biopsies.

The activity of the mitochondrial respiratory chain (COX/SDH-activity ratio) was depressed in ILD compared with control biopsies (p < 0.001). Furthermore, the COX/SDH-activity ratio was negatively correlated with the pulmonary MDA (r = −0.84, p < 0.001; Fig. 1a).

The COX2/COX4-ratio was depressed in the lung biopsies from ILD-patients (median 0.6) in comparison with biopsies from controls (median 2.2; p < 0.001; Table 1). The COX2/COX4-ratio was moderately correlated with respiratory chain function in terms of the COX/SDH-activity ratio (r = 0.39, p < 0.001; Fig. 1b) and negatively correlated with the pulmonary MDA content (r = −0.33, p = 0.005; Fig. 1c).

On the genetic level, wild-type mtDNA copy numbers per cell were slightly elevated in ILD-patients but total mtDNA amounts did not differ statistically between patients and controls (p = 0.088; Table 1). The common mtDNA deletion was present at low levels in some control lungs (median percentage of mtDNA deletions 0%, Table 1). ILD-patients in contrast harboured significantly higher levels of the common deletion (median percentage of mtDNA deletions 17%, p < 0.001 *vs* controls, see also Supplementary Fig. 1). In all lung biopsies, the amounts of mtDNA deletions were negatively correlated with the COX/SDH-activity ratio (r = −0.59, p < 0.001; Fig. 1d) and the COX2/COX4-ratio (r = −0.49, p < 0.001; Fig. 1e). Conversely, the amounts of mtDNA deletions correlated positively with the MDA-content (r = 0.69, p < 0.001; Fig. 1f).

In summary, these data indicate that fibrotic lungs acquired high levels of dysfunctional mtDNA molecules, in association with depressed synthesis of mtDNA-encoded respiratory chain subunits, respiratory chain dysfunction and enhanced ROS-formation.

### Comparison of upper and lower lung biopsies

**Figure 2:**
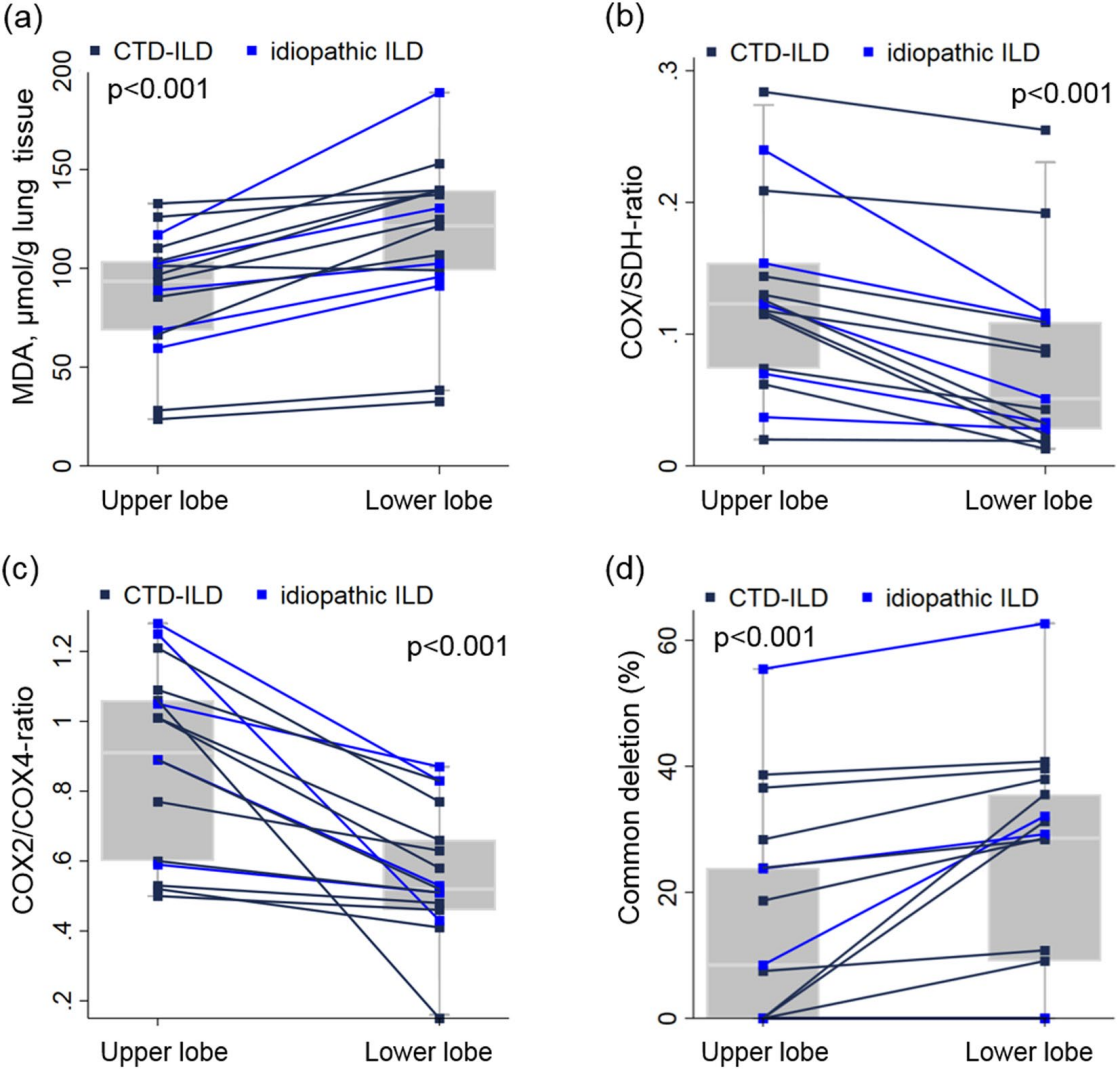
Comparison of lung biopsy sites (upper *vs*. lower lobes) for (**a**) ROS-formation, (**b**) mtDNA encoded respiratory chain function, (**c**) mtDNA-encoded protein, (**d**) mtDNA mutations in those 17 ILD-patients in whom simultaneous biopsies were performed. Lines connect measurements from the same patient. Boxes represent the 25th, 50th and 75th percentiles; whiskers define the lowest and highest data point within 1.5 times the interquartile range.

Among those 17 patients of whom we had simultaneous biopsies from upper and lower lobes, the median MDA content was 30% higher in the lower lobes (121.5 μmol MDA/g tissue, IQR 99.0–139.5) than in the upper lobes (93.4 μmol MDA/g tissue, IQR 68.6–103.5; Fig. 2a). The median COX activity, normalized for the activity of SDH (which is solely encoded by nDNA) was however 41% lower in the basal lobe biopsies compared to upper lobe biopsies (p < 0.001; Fig. 2b). The COX2/COX4-ratio was 35% lower (median COX2/COX4-ratio 0.52, IQR 0.46–0.66) in the biopsies from lower lobes as compared to their upper lobe counterparts (0.91, IQR 0.60–1.06, p < 0.001; Fig. 2c). Whereas the amount of wild-type mtDNA copies did not differ between the two biopsy sites (median 434, IQR 301–542 copies for upper lobe biopsies and 441, IQR 338–606 copies in lower lobe biopsy sites, p = 0.83), the median amounts of the common deletion were elevated by 53% in lower compared to upper lobes (p < 0.001; Fig. 2d).

These results demonstrate that in each of the examined individuals, mitochondrial alterations and ROS-formation are enhanced in the lower lobe regions, in agreement with the typical apicobasal gradient of fibrosis in ILD.

### Other predictors of ROS-formation and mitochondrial lesions

There was no association of any of these mitochondrial parameters and gender within patient or controls. The patients’ age was not associated with pulmonary MDA-levels (β = 0.29, 95%CI −0.7 to 1.3, p = 0.58), the COX/SDH-ratio (β = −0.0005, 95%CI −0.002 to 0.001, p = 0.63) and mtDNA copy numbers (β = −0.054, 95%CI −2.6 to 2.5, p = 0.97) in all biopsies, when accounting for patient groups. There was however a positive association between the frequency of the common mtDNA deletion and age (β = 0.31, 95%CI −0.007 to 0.6, p = 0.055 per 1-year increase in age) as well as a negative correlation between the COX2/COX4-ratio and age (β = −0.009, 95%CI −0.017 to −0.001, p = 0.022 per 1-year increase in age). Smoking status was not associated with any of the assessed parameters (Table 2).

**Table 2:** Comparison of lung parameters in all lung biopsies by smoking status separate for lung fibrosis patients and controls.

	Never smokers	Previous smokers	Current smokers	p-value
**Cases**				
MDA, μmol/g lung tissue	98 (89–125)	103 (94–131)	100 (97–102)	0.52
COX/SDH-ratio	0.12 (0.09–0.14)	0.07 (0.03–0.11)	0.11 (0.10–0.13)	0.17
mtDNA, copies/pulmonary cell	385 (325–484)	414 (365–487)	426 (365–487)	0.92
COX2/COX4-ratio	0.81 (0.57–1.01)	0.64 (0.51–0.83)	0.73 (0.47–0.99)	0.28
common deletion, % of mtDNA molecules	8.0 (0–31)	24.3 (0–32)	27.1 (22–32)	0.21
**Controls**				
MDA, μmol/g lung tissue	115	91 (66–102)	89 (87–91)	0.29
COX/SDH-ratio	0.14	0.12 (0.11–0.19)	0.15 (0.12–0.18)	0.82
mtDNA, copies/pulmonary cell	262	342 (304–504)	327 (315–339)	0.47
COX2/COX4-ratio	2.10	2.20 (1.98–2.44)	1.98 (1.79–2.18)	0.45
common deletion, % of mtDNA molecules	12.7	0 (0–14)	0 (0–0)	0.41

Presented are medians and interquartile ranges.

COX2/COX4, cytochrome c-oxidase subunit 2 protein normalized for cytochrome c-oxidase subunit 4 protein; COX/SDH-ratio, cytochrome c-oxidase/succinate dehydrogenase activity ratio; MDA, malondialdehyde; mtDNA, mitochondrial DNA.

When comparing biopsies from idiopathic ILD with those of CTD-associated ILD, there was no difference with respect to MDA-levels (p = 0.65), the COX/SDH-ratio (p = 0.93), the COX2/COX4-ratio (p = 0.61), mtDNA copy numbers (p = 0.22) and the prevalence of the common deletion (p = 0.42). Similarly, there were no differences in mitochondrial parameters between lungs from patients with SSc and those with other CTDs (data not shown).

## Discussion

The aim of this study was to investigate the role of mitochondrial dysfunction in the pathogenesis of both, idiopathic and CTD-related ILD and the relentless progression of ILD over time. We demonstrate in human lung biopsies, that fibrosis is associated with ROS-formation, mitochondrial dysfunction and the development of large-scale deletions in mtDNA as identified in our previous study in the bleomycin model of lung fibrosis^14^. The mitochondrial lesions are unexplained by smoking status and were more protuberant in the lower than in the upper lobes, in line with the typical apicobasal gradient of fibrotic manifestations. Our data indicate that the respiratory impairment is associated with a selective impairment of mitochondrial, but not nuclear encoded respiratory chain subunits.

An aberrant response to recurrent insults is an important pathophysiological mechanism in the development of ILD^1,15^. Substantial evidence suggests that ROS of pulmonary origin drive the fibrotic process^4,5,7^. In search of a possible role of mitochondria in ROS-generation, data from the bleomycin model of lung fibrosis suggest that mtDNA insults could perpetuate ROS-formation and mitochondrial lesions^14^. The important contribution of ROS to lung fibrosis is also demonstrated by the observation that the absence of ROS protects against bleomycin mediated lung fibrosis^16^.

In the current study, we demonstrate that substantial mitochondrial dysfunction is likely to represent a significant source of ROS in human lung fibrosis. mtDNA and respiratory chain insults could self-perpetuate each other and promote the relentless progression and subsequent age-related acquisition of lung fibrosis. Whereas it has been demonstrated that ROS can activate TGFβ1, a profibrotic cytokine^8,17,18^, it is only poorly understood, if mitochondrial dysfunction may also promote fibrosis by other mechanisms^18^, i.e. by directly triggering apoptosis in pulmonary tissues^7^. Recent studies in an *in vivo* model of acute lung injury have demonstrated a diminished synthesis of alveolar adenosine triphosphate in conjunction with mitochondrial dysfunction and a protective effect of normal mitochondrial bioenergetics^19^. Lastly, oxidative mtDNA damage impairs cell viability and promotes netosis which is associated with an extracellular release of mtDNA molecules as damage-associated molecular pattern and predictor of death in lung fibrosis^20,21^.

We have to acknowledge, that our patient and control populations were not age matched leading to an older control group. Age in our study was however associated with a well-known higher prevalence of mtDNA deletions and depressed mtDNA encoded respiratory chain function^22^. The older control group would be as a consequence of age rather be expected to have more mitochondrial lesions than the ILD patient group. We, however, found the opposite. Therefore, due to the age disparity between our patient and control group, we may have rather underestimated than overestimated the role of mitochondrial dysfunction in ILD identified in our study. Smoking is also a known inducer of oxidative stress^23^. Disparities of smoking behaviour between control and ILD-patients, are however unlikely to explain the differences of malondialdehyde content or mitochondrial function observed between control and ILD-patients, as smoking was not associated with any parameter. Lastly, it should be mentioned that we were unable to retrieve information of the patients’ treatment prior to the acquisition of the biopsies. It is however unlikely that the subjects under study had significant amounts of radiotherapy or chemotherapy before the lung biopsy, as the diagnosis triggering such therapeutic interventions usually require biopsy confirmation.

Our results do not provide proof of a causal role of mitochondrial dysfunction in ILD and we have to acknowledge that we do not know which of the various pulmonary cells accounts for our findings. We have previously identified an architectural distortion of mitochondria in several pulmonary cell types, including alveolar epithelial cells in ultrastructural studies of the bleomycin model of lung fibrosis^14^. Others have demonstrated accumulation of dysmorphic and dysfunctional mitochondria in alveolar epithelial cells, fibroblasts and macrophages of idiopathic ILD-patients^24^. Preclinical models have demonstrated that alveolar epithelial cells acquire oxidative mtDNA lesions and that alveolar epithelial cell dysfunction in response to such injury is fundamental in the development of ILD^13,25^. Lastly, it was shown that an enhancement of mtDNA repair capability in lung endothelial cells is able to antagonize their ROS-mediated mtDNA damage, cytotoxicity, and propensity to undergo apoptosis^20^.

In summary, our data demonstrate a profound distortion of mitochondrial function in idiopathic and CTD-associated ILD that could account for a self-perpetuating release of endogenous ROS as an important contributor of fibrosis. These findings support the development of pharmacologic strategies aimed at enhancing pulmonary mtDNA repair and mitochondrial bioenergetics.

## Methods

### Patients and lung biopsies

**Figure 3:**
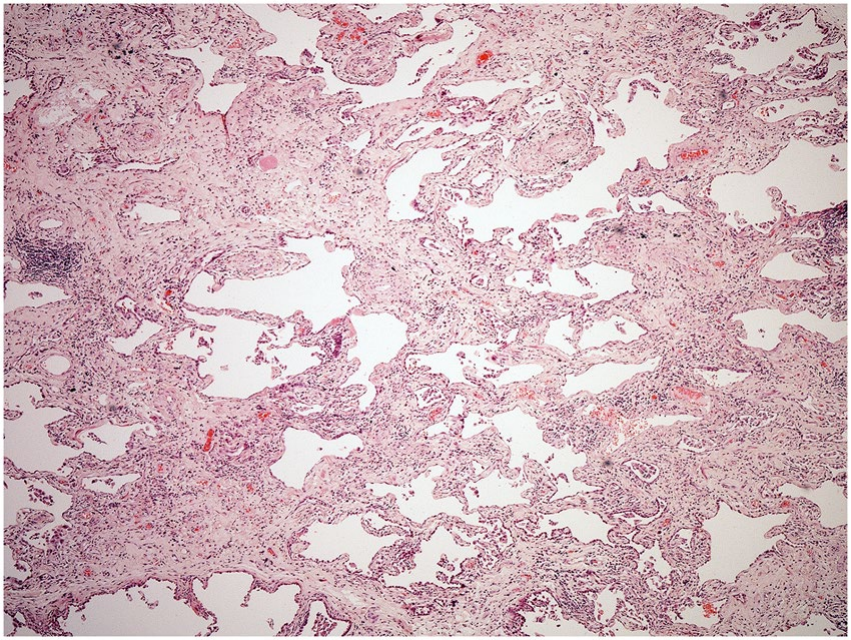
Fibrotic non-specific interstitial pneumonia (NSIP). The biopsy shows diffuse homogenous interstitial fibrosis associated with a mild non-specific chronic inflammatory cell infiltrate. Neither fibroblastic foci nor honeycomb change is seen (haematoxylin and eosin stain, x40).

Adult patients of the Royal Brompton Hospital, London, UK with either idiopathic or CTD-related ILD were included in the study. All showed a histological pattern of fibrotic non-specific interstitial pneumonia (Fig. 3), one with coexistent organising pneumonia in the lower lobe. Control samples were taken from background lung of resections for lung cancer without interstitial fibrosis. All samples were surgical lung biopsies.

All patients signed an informed consent for the research use of their samples, and the study was conducted in accordance with the 1975 Declaration of Helsinki, as revised in 2008.

### ROS products in lung tissue

The generation of ROS is an unavoidable by product of mitochondrial electron-transport. We spectrophotometrically quantified malondialdehyde (MDA) as one of the products of ROS-mediated lipid peroxidation by probing for thiobarbituric acid reactive material^26^.

### Activity of mitochondrial respiratory chain complexes

The enzymatic activity of cytochrome c-oxidase (COX, also known as respiratory chain complex IV) was assessed in lung extracts by spectrophotometry, as described^27^. The COX subunits are encoded in part by nuclear DNA (nDNA) and in part by mtDNA. We similarly quantified the enzymatic activity of succinate dehydrogenase (SDH) as the respiratory chain complex II which is entirely encoded by nDNA^27^.

### MtDNA-encoded respiratory chain protein

The subunit-2 of cytochrome c-oxidase (COX2) is encoded by mtDNA, while subunit-4 of cytochrome c-oxidase (COX4) is encoded by nDNA. A low COX2/COX4-ratio is therefore an indicator of an impaired synthesis of mtDNA-encoded respiratory protein. Both COX subunits were quantified in lung tissues by immunoblot densitometry (Quantum ST4v16.03, Vilber Lourmat, Marne-la-Vallee, France)^28^.

### MtDNA-content

Genomic DNA was extracted from lung tissues with a DNA isolation kit (QIAamp, Qiagen, Germany). MtDNA copy numbers were quantified on a 384 well plate by quantitative PCR using the LightCycler® 480 Real-Time PCR System (Roche, Germany). 10 μl reactions contained 5 μl of SYBR Green I Master mix (Roche, Germany) 10 ng DNA template and 0.5 μM of primers (5′-ACCAATAGCCCTGGCCGTAC-3′and 5′-GGTGGCGCTTC-CAATTAGGT-3′)^29^. All samples were run in triplicate. MtDNA copy numbers were normalized for nDNA copy numbers of glycerol aldehyde phosphate dehydrogenase. Absolute mtDNA and nDNA copy numbers were measured by employing a standard curve with serial dilutions of mtDNA and nDNA plasmids. The mtDNA copy number per pulmonary cell was calculated as the number of mtDNA copies per two nDNA copies.

### Quantification of the common mtDNA-deletion

During mtDNA replication, sequence base pairs may be deleted by slipped mispairing between direct nucleotide sequence repeats^30^. In humans, a 4977 base-pair deletion is the most prevalent result of slipped mispairing and therefore termed “common” deletion. We probed for the common mtDNA deletion by amplifying 100 ng of pulmonary DNA in a PCR reaction with extradeletional primers (5′-TAA TTC CCC TAA AAA TCT TTG AAAT-3′ and 5′-AAC CTG TGA GGA AAG GTA TTC CTGC-3′)^31^. By means of a short extension cycle (30 seconds), the deleted molecule was preferentially amplified as a 324 base-pair product (Supplementary Fig. 1) and confirmed by sequencing (MWG Biotech, Germany) to represent the common mtDNA deletion. The deletion was quantified on agarose gels with a standard curve of PCR products from known ratios of deleted and wild type mtDNA molecules as described elsewhere^31^.

### Statistics

Categorical variables are presented as frequencies/percentages and continuous variables as means/standard deviations (SD) or medians/interquartile ranges (IQR). T-tests or Wilcoxon–Mann–Whitney tests were applied for across group comparisons in unpaired, non-clustered data and Somers’ D was used when all biopsies were included accounting for the biopsy clustering on the patient level. Paired Wilcoxon-signed-rank tests were used to assess differences of the measures in the upper *vs* lower lung biopsies. Correlations between continuous measures are stated as Pearson’s correlation coefficient. Linear regression analysis was applied to adjust for possible confounders. All data analyses were performed with Stata/IC 13.1 (StataCorp, College Station, USA).

### Prior abstract publication/presentation

This study has been presented at the 2017 American College of Rheumatology (ACR) congress held in San Diego and at the Annual Congress of the SGR-SSR and HPR Symposium as a poster and at the 2018 European League against Rheumatology (EULAR) congress held in Amsterdam as an oral presentation.

### Ethical approval

Utilisation of archival frozen samples was approved by the Royal Brompton & Harefield NHS Foundation Trust (ethics approval reference EKBB 353/11).

## Supplementary information

Supplementary figure 1

## Data Availability

The datasets analysed during the current study are available from the corresponding author on reasonable request.
