# Supplementary material for: Mitochondrial DNA mutations and respiratory chain dysfunction in idiopathic and connective tissue disease-related lung fibrosis
Source: Sci Rep. 2019 Apr 2;9:5500. doi: 10.1038/s41598-019-41933-4 (PMC6445113; doi:10.1038/s41598-019-41933-4)

Mitochondrial DNA mutations and respiratory chain dysfunction in idiopathic and connective tissue disease-related lung fibrosis

Veronika K. Jaeger[1], Dirk Lebrecht[2,3,], Andrew G. Nicholson[4,5], Athol Wells[5,6], Harshil Bhayani[7], Amiq Gazdhar[8], Michael Tamm[9], Nils Venhoff[2], Thomas Geiser[8], and Ulrich A. Walker[1]*

[1] Department of Rheumatology, University Hospital Basel, Basel, Switzerland

[2] Department of Rheumatology and Clinical Immunology, Medical Center - University of Freiburg, Faculty of Medicine, University of Freiburg, Freiburg, Germany

[3] Department of Pediatrics and Adolescent Medicine, Division of Pediatric Hematology and Oncology, Medical Center, Faculty of Medicine, University of Freiburg, Freiburg, Germany

[4] Department of Histopathology, Royal Brompton Hospital, Royal Brompton and Harefield NHS Foundation Trust; London, UK

[5] National Heart and Lung Institute, Imperial College, London, UK

[6] Interstitial Lung Disease Unit, Royal Brompton Hospital, Royal Brompton and Harefield NHS Foundation Trust, London, UK

[7] Royal Brompton and Harefield NHS Foundation Trust; London, UK

[8] Department of Pulmonary Medicine, University Hospital Bern, Bern Switzerland

[9] Department of Pneumology, University Hospital Basel, Basel, Switzerland

Supplementary figure 1: Representative image of an agarose gel detecting the common mtDNA deletion. Lane 1: 100bp DNA ladder as a size marker (Sigma). Lane 2: Negative control plasmid without mtDNA insert. Lane 3: Healthy control DNA. Lanes 4-6: Detection of common deletions in DNA from patients with interstitial lung disease in different lung areas (lower lobe, upper lobe, and upper lobe, respectively). Lane 7: Positive control (plasmid containing the common mtDNA deletion).

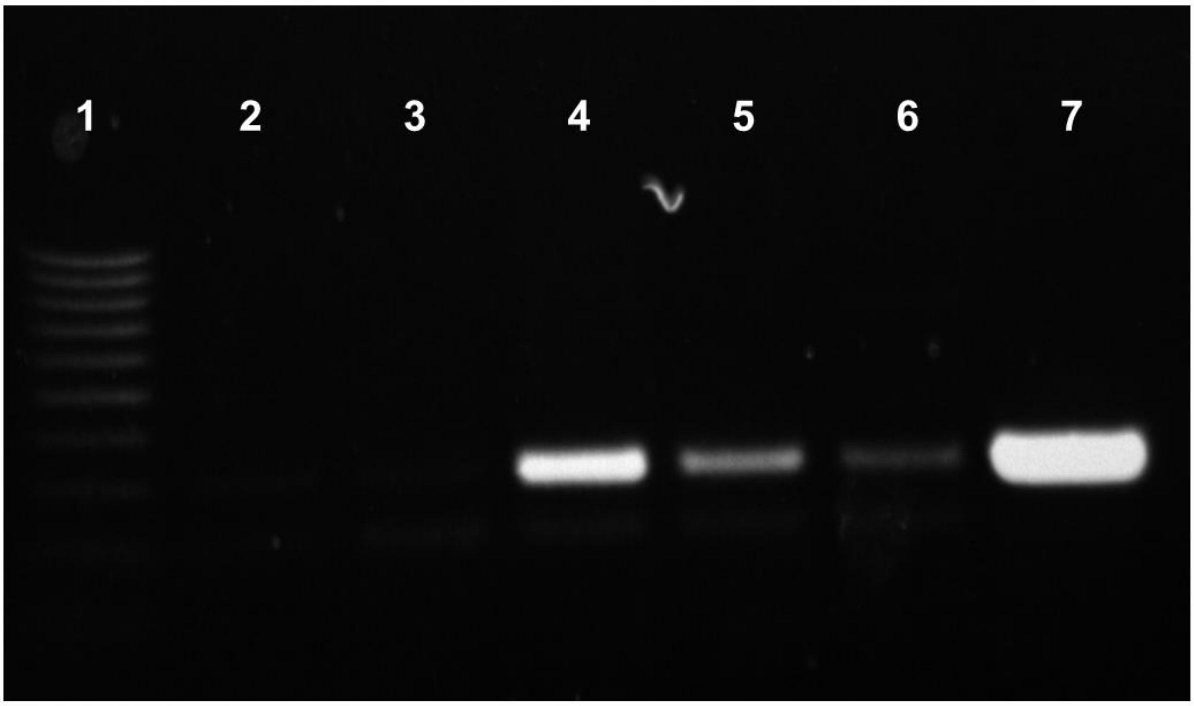

Supplement: Supplementary file 1 — Supplementary figure 1 [file 41598_2019_41933_MOESM1_ESM.pdf]
